# Irritancy and spatial repellency efficacy of repellent-treated fabrics against *Aedes aegypti* (L.) (Diptera: Culicidae) in an excito-repellency system

**DOI:** 10.1371/journal.pone.0336729

**Published:** 2026-02-26

**Authors:** Alex Ahebwa, Jeffrey Hii, Theerachart Leepasert, Jirod Nararak, Monthathip Kongmee, Theeraphap Chareonviriyaphap

**Affiliations:** 1 Department of Entomology, Faculty of Agriculture, Kasetsart University, Bangkok, Thailand; 2 Research and Lifelong Learning Center for Urban and Environmental Entomology, Kasetsart University Institute for Advanced Studies, Kasetsart University, Bangkok, Thailand; 3 College of Public Health, Medical and Veterinary Sciences, James Cook University, North Queensland, Australia; 4 Department of Chemistry, Faculty of Science, Kasetsart University, Bangkok, Thailand; 5 Department of Entomology, Faculty of Agriculture, Kasetsart University, Nakhon Pathom, Thailand; 6 Royal Society of Thailand, Bangkok, Thailand; Chiang Mai University Faculty of Agriculture, THAILAND

## Abstract

Mosquito-borne diseases remain a major public health challenge, driving the need for affordable and scalable vector control tools. In this study, an excito-repellency system was used to evaluate the contact irritancy and spatial repellency potential of two low-cost repellent-treated fabrics consisting of Calico (100% cotton) and Jute (hessian), and a standard treated bed net polyester (BNP), against a laboratory strain of *Aedes aegypti* (L.). Fabric swatches (15 x 17.5 cm) were treated with six concentrations of transfluthrin and metofluthrin, and five of permethrin. Behavioral responses were measured via chamber escape over 30 minutes using 60 unfed female mosquitoes per treatment. Chemical retention was assessed using gas chromatography–mass spectrometry (GC-MS). Calico elicited the highest mosquito escape (Odds ratio, OR = 3.12) followed by Jute (OR = 1.74) relative to BNP. Transfluthrin (OR = 5.45) produced the highest escape among insecticides, whereas low dose treatments resulted in more escape (OR = 1.23) than high dose applications. Non-contact chambers elicited more escape (OR = 1.87) than the contact chambers, indicating stronger spatial repellency than contact irritancy. Toxicity was most pronounced with metofluthrin across fabrics (Mortality: OR = 32.27), particulary on BNP which corresponded with reduced escape. GC-MS results showed stable permethrin retention across fabrics, whereas transfluthrin retention varied significantly between Calico and BNP after 24 h drying. These findings highlight the importance of fabric–insecticide compatibility and the influence of exposure method, dose, and chemical volatility on repellent efficacy. Future studies could investigate chemical interactions between repellents and fabrics to clarify their combined effects.

## Introduction

*Aedes aegypti* is the most widely distributed mosquito vector globally, responsible for transmitting arboviruses of major public health concern, including dengue, chikungunya, Zika, and yellow fever [1,2]. The rising incidence of these diseases is driven by climate change, urbanization, global travel, and expanding trade networks [3,4]. In the absence of widely accessible vaccines and reliable treatment for many arboviral infections, mosquito bite prevention and vector control remain the cornerstone of disease mitigation [5].

Insecticide-treated nets and clothing have been very effective against malaria vectors [6,7], however, their efficacy against diurnal vectors like *Ae. aegypti* is inconsistent [8]. Given the limited use of nets during daytime, insecticide-treated clothing offers a practical alternative, especially in occupational and recreational settings [9,10]. Their integration into public health settings may be facilitated by occupational norms, such as protective clothing worn by rubber tappers [11] and the availability of guidelines for home treatment [12]. Recent studies have explored a range of textile substrates, including natural fibers like cotton and jute, and synthetic blends such as polyester and nylon, each with distinct insecticide retention and repellent profiles [13–17]. Cotton and jute are of particular interest as low-cost and widely available materials that may serve as carriers of repellent compounds [12,14,18]. Innovations in mosquito-repellent textiles now extend to outdoor wear, uniforms, and beddings, with efficacy shaped by fabric porosity, weave density, and treatment method [12]. Despite these advances, comparative evidence on the performance of different fabric-insecticide combinations remains limited.

Permethrin is the most commonly used insecticide used in insecticide-treated clothing and acts through contact-mediated irritancy, disrupting mosquito landing behaviour [19,20]. When combined with a topical repellent, near-complete protection has been achieved [21–23]. However, protection may be reduced where skin remains exposed or in regions with insecticide-resistant vector populations. In contrast, volatile pyrethroid spatial repellents (VPSRs) such as transfluthrin and metofluthrin, offer an added layer of protection with olfactory-mediated repellency that bypasses traditional resistance mechanisms [5,24,25]. Despite this promise, empirical validation of VPSR-impregnated fabrics remains limited. It is reasonably difficult to measure repellency under field conditions or even semifield conditions given the nature of the VPSRs [26–28]. The excito-repellency system distinguishes between spatial repellency (escape without physical contact) and contact irritancy (escape after contact)*,* thereby providing a controlled environment for quantifying mosquito escape behaviors under laboratory conditions [29,30].

This study, therefore, used an excito-repellency system to compare the behavioural responses of *Ae. aegypti* to two low-cost fabrics—Calico [100% cotton] and Jute [hessian/burlap]—impregnated with transfluthrin, metofluthrin or permethrin. Their performance was compared to standard bed net polyester (BNP). The retention of transfluthrin and permethrin in the fabrics before and after drying was quantified using the gas chromatography – mass spectrometry (GC-MS).

## Materials and methods

### Mosquito rearing

*Aedes aegypti*, a laboratory-susceptible USDA strain that has been maintained for over 20 years at Kasetsart University, Bangkok, Thailand, was used for all bioassays. Rearing followed Ahebwa et al [14]: 26 ± 2 °C, 70 ± 10% relative humidity, and a 12:12 h light:dark photoperiod. Eggs were hatched in tap water (2 L/tray), and larvae were reared at a density of 250 per tray, fed daily on commercial food pellets (PondMax, Australia; 2 pellets ≈100–150 mg per tray). Pupae were transferred to 30 × 30 × 30 cm cages for adult emergence. Adults were sustained on 10% sugar solution through cotton sticks; females were blood-fed twice weekly using CPDA-1 preserved human blood (from Thai Red Cross Society) via an artificial membrane feeding system. Moistened filter papers were introduced into each cage to encourage oviposition, and dried eggs were stored for colony maintenance. Unfed, 3–5 days-old female adults were selected for bioassays.

### Volatile pyrethroid preparation and dose-ranging

Technical-grade transfluthrin (97.9%; CAS 118712-89-3) and metofluthrin (96.4%; CAS 240494-70-6) were obtained from Earth (Thailand) Co. Ltd., Bangkok. Permethrin (94%) was provided by the Ministry of Public Health, Thailand. Preparingstock solutions, analytical-grade acetone (Avantor Performance Materials, Inc., Allentown, PA, USA) was used as an organic solvent, and silicone oil (Dow Corning1556, Dow Chemical Co., Midland, MI, USA) as a carrier at a 0.96:1.95 ratio [14]. Preliminary dose-range optimization was conducted using calico fabric swatches (15 x 17.5 cm) impregnated with 3 mL insecticide solution. Reference discriminating concentrations were 0.06824% w/v for transfluthrin and metofluthrin [31] and 0.4% w/v for permethrin [32]. Subsequently, six transfluthrin (and metofluthrin) concentrations (1.0–31.3 mg/m^2^) and five for permethrin (0.25–4 g/m^2^) were prepared by serial dilution.

### Fabric preparation and treatment

Three fabric types were sourced from commercial suppliers in Bangkok, Thailand comprising Calico (100% cotton), Jute (100% burlap/hessian yarn fabric), and BNP. Prior to use, all fabrics were screened for insecticide contamination using WHO cone bioassays with 200 laboratory-susceptible *Ae. aegypti* females. Any fabric yielding >5% mortality at 24 h was excluded. Each fabric was cut into eight 15 x 17.5 cm swatches: four for contact and four for non-contact chambers of the excito-repellency system. Four untreated control pieces were reused across bioassays. Fabrics were treated with 3 mL of insecticide solution using calibrated 10 mL glass pipettes, following WHO guidelines [33]. Control pieces received an equivalent volume of solvent mixture. Treated fabrics were air-dried for 24 h at 26 ± 4 °C, 70 ± 10% relative humidity under a 12:12 light:dark photoperiod. Insecticide-treated fabrics were discarded after each test.

### Excito-repellency test system

**Fig 1:**
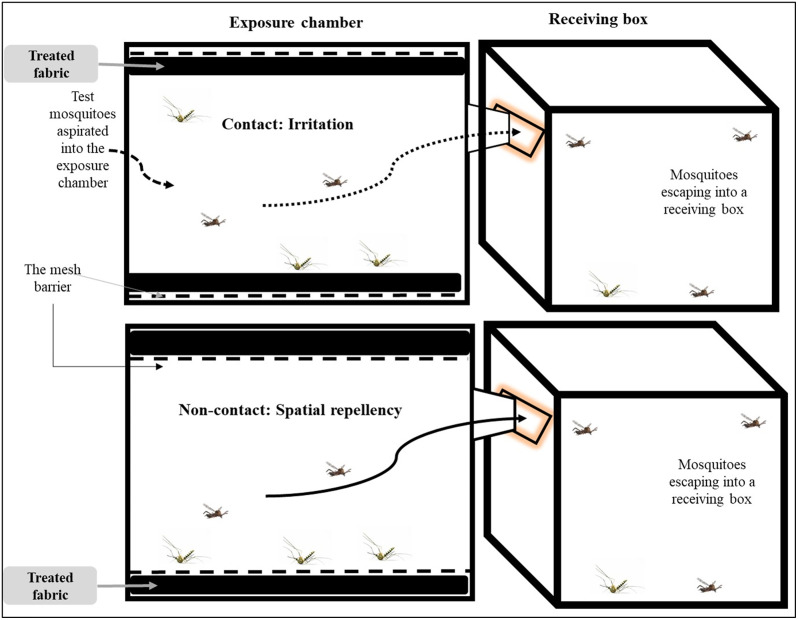
Cross-sectional view of the excito-repellency system. The contact and non-contact exposure chambers are shown in the upper and lower left panels, respectively. Each chamber is connected to a receiving box on the upper and lower right panels.

The improved excito-repellency system, based on the original design by Chareonviriyaphap et al [34], with modifications described in our recent studies [35,36], distinguishes between contact irritancy and non-contact spatial repellency (Fig 1). In this study, repellent-treated fabrics replaced the standard Whatman No. 1 filter papers. Each bioassay consisted of a single fabric type, exposure mode, and insecticide concentration tested against 60 adult female *Ae. aegypti* in four replicates of 15 mosquitoes. After a 3-min acclimation period, exit doors were opened into adjacent receiving boxes. Escaping mosquitoes were recorded over 30 min. Escaped and non-escaped mosquitoes were transferred to labeled, net-covered paper cups and provided 10% sugar solution. Knockdown was assessed 1 h after each bioassay while mortality was recorded 24 h later. Room conditions were maintained at 25 ± 2 °C and 70 ± 5% relative humidity throughout the experiment. Chambers were cleaned with acetone between tests and fully submerged in acetone overnight between insecticides, then air-dried for 24 h before reuse.

### Chemical retention analysis

GC-MS was employed to assess transfluthrin and permethrin retention in Calico, Jute, and BNP fabrics after 1 h and 24 h drying intervals, under controlled laboratory conditions (25 ± 2 °C; 70 ± 10% relative humidity). Previous findings suggest that transfluthrin concentrations decline over time, potentially reducing efficacy [37]. Following impregnation, each insecticide was applied to a single 15 × 17.5 cm fabric piece. Three 4 cm² swatches were excised at each time point for chemical extraction. Swatches were soaked overnight in analytical-grade methanol and sonicated for 10 min; extracts were transferred to glass vials for GC-MS analysis.

A QP2020 gas chromatography system (Shimadzu, Japan) with SH-Rxi-5Sil MS column (30 m × 0.25 mm i.d., 0.25 µm film) was used. Helium was used as the carrier gas at 1.2 mL/min with a split ratio of 1:10. The injector was maintained at 230 °C. Oven temperature was programmed from 50 °C (initial) to 220 °C at a ramp rate of 4 °C/min. Selected ion monitoring was employed for compound detection. Target ions were m/z 163 and 91 for transfluthrin [38,39], and m/z 183, 163, and 165 for both cis- and trans-permethrin [40]. Metofluthrin was excluded from this analysis due to logistical limitation.

### Data analysis

Raw data were entered in Microsoft Office Excel (Windows 11) and imported into RStudio version 4.1.0 [41] for statistical analysis. As the analysis focused on the efficacy of the treated fabrics, insecticide doses were categorized as low (first three) or high (subsequent) based on escape patterns observed in preliminary trials. This dose classification allowed for consistent comparison across insecticides and fabrics, while retaining operational relevance for field application. Data manipulation and cleaning were accomplished using the *tidyr* package [42]. The escape rates were adjusted with the paired controls to set the controls at zero based on the Abbott’s formula [43].


$$\text{Corrected escape rate }(\;\%\;)=\frac{\left(\%\text{ escape in test }-\;\%\text{ escape in control}\right)}{\text{100 }-\;\%\text{ escape in control }}\text{X 100}$$

To model the probability of mosquito escape, a generalized linear mixed model (GLMM) was fitted using the glmer function from the lme4 package. The response variable—number of mosquitoes released per replicate—was binary (escaped vs not escaped). Fabric type, exposure mode, insecticide type, and dose category were treated as fixed effects, and only replicates as random effects. Insecticide x fabric interactions were modeled to assess whether the effect of insecticide type on escape behavior varied across fabric types. Model fit was evaluated using Akaike Information Criterion, and statistical significance of predictors was assessed via likelihood ratio tests and Wald statistics [44]. Model outputs—including estimated coefficients, standard errors, odds ratios, 95% confidence intervals (CIs), and P-values—were summarized in tabular format to facilitate interpretation of fixed effects and their relative influence on escape behavior. 

To assess the escape patterns, Kaplan-Meier curves were generated using the survfit function from the survival package, providing non-parametric estimates of retention probabilities (number of mosquitoes that did not escape) over time, with escape as the event. Escape probability was calculated relative to the total number of mosquitoes recovered at the end of each bioassay. Visualizations were produced with ggsurvplot from the survminer package. Differences among groups were evaluated using the log-rank test, with statistical significance defined as *p* < 0.05. Right-censored data (i.e., mosquitoes that did not escape within the observation period) were appropriately accounted for [45].

## Results

### Irritancy and spatial repellency

Mosquito escape behavior was analysed using GLMM and Kaplan-Meier curves, with escape treated as the event. Irritancy was assessed from escape in contact trials, while spatial repellency was inferred from escape in non-contact trials.

All main effects significantly increased the odds of mosquito escape (*P* < 0.001), except for insecticide x fabric interaction terms. Relative to metofluthrin, transfluthrin produced significantly elevated odds of escape (OR = 5.45; 95% CI: 3.96–7.50), statistically similar to permethrin (OR = 3.26; 95% CI: 2.32–4.58). Amongst fabrics, Calico significantly increased odds of escape (OR = 3.12; 95% CI: 2.24–4.34), followed by Jute (OR = 1.74; 95% CI: 1.22–2.47), relative to BNP. Notably, the insecticide x fabric interaction terms revealed contrasting effects for Calico: treatment with transfluthrin showed reduced odds of *Ae. aegypti* escape (OR = 0.51; 95% CI: 0.35–0.77), whereas treatment with permethrin showed increased odds (OR = 1.45; 95% CI: 0.95–2.20). The odds of escape were significantly higher in non-contact trials (OR = 1.87; 95% CI: 1.65–2.11) than contact trials, and at low dose level (OR = 1.23; 95% CI: 1.09–1.38) than high dose (Table 1).

**Table 1:** GLMM estimates of *Aedes aegypti* escape upon exposure to insecticide-treated fabrics.

Variable		Estimate	SE	OR	95% CI	p-value
(Intercept)		1	–	–	–	–
Fabric	Calico	1.137	0.169	3.117	2.238–4.341	***
	Jute	0.552	0.181	1.736	1.218–2.474	**
Test type	Non-contact	0.624	0.062	1.866	1.654–2.105	***
Dose category	Low	0.205	0.061	1.227	1.088–1.384	***
Insecticide	Permethrin	1.182	0.173	3.259	2.322–4.576	***
	Permethrin x Calico	0.370	0.214	1.448	0.953–2.200	0.083
	Permethrin x Jute	−0.323	0.228	0.724	0.463–1.130	0.155
	Transfluthrin	1.696	0.163	5.451	3.961–7.501	***
	Transfluthrin x Calico	−0.664	0.203	0.514	0.346–0.766	**
	Transfluthrin x Jute	−0.283	0.213	0.754	0.496–1.145	0.185

OR = Odds ration; SE = Standard error; CI = confidence interval. ORs are relative to the reference levels; OR > 1 indicates increased odds, OR < 1 indicates reduced odds. Interaction terms (e.g., Permethrin x Calico) reflect conditional effects.

**Fig 2:**
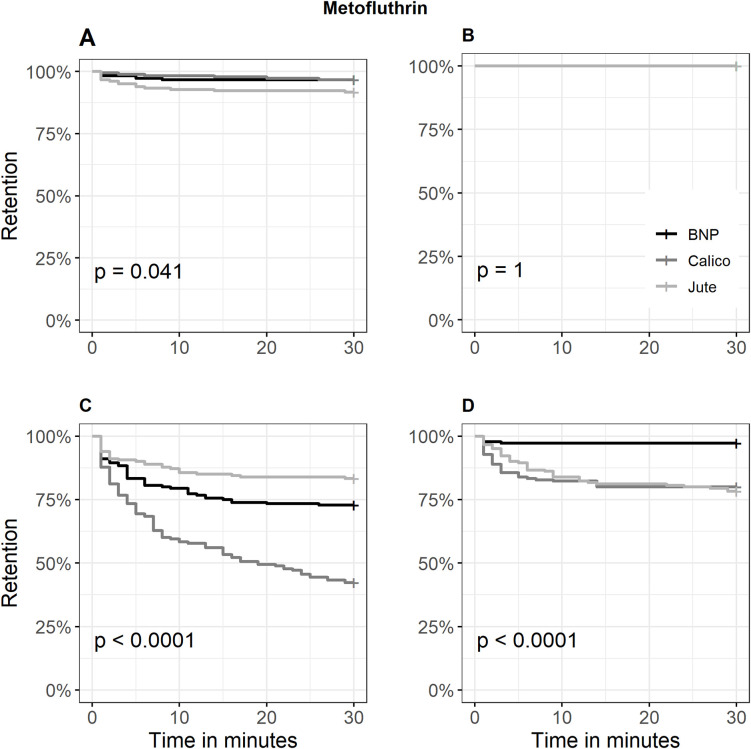
Kaplan–Meier curves for the retention probability of *Aedes aegypti* across three metofluthrin-treated fabrics. A = Contact retention probability at low dose; B = Contact retention probability at high dose; C = Non-contact retention probability at low dose; D = Non-contact retention probability at high dose; BNP = Bed net polyester fabric. The curves represent the proportion of mosquitoes remaining in the treated chamber (retention) over time, with escape treated as the event. Statistical significance was assessed using the log-rank test.

**Fig 3:**
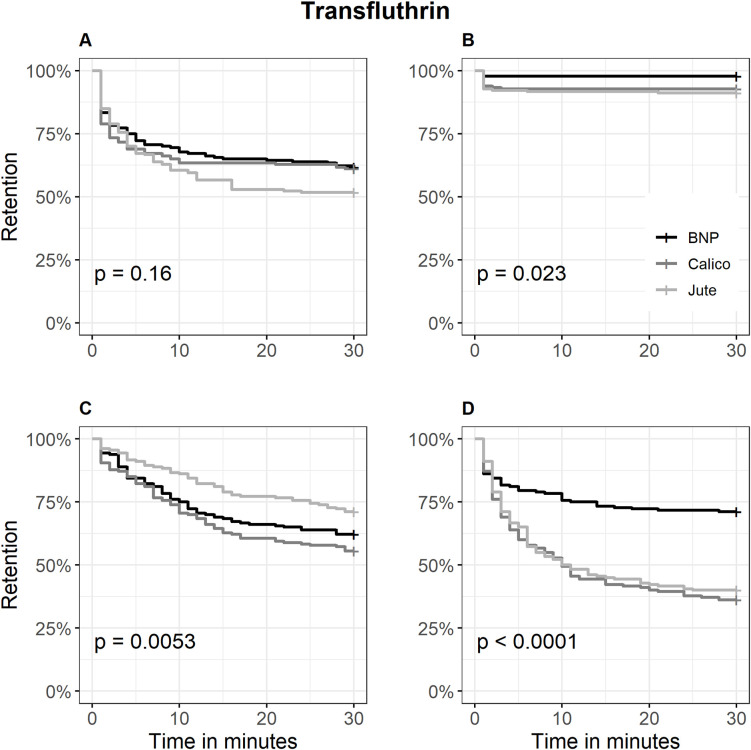
Kaplan-Meier curves for the retention probability of *Aedes aegypti* across three transfluthrin-treated fabrics. A = Contact retention probability at low dose; B = Contact retention probability at high dose; C = Non-contact retention probability at low dose; D = Non-contact retention probability at high dose; BNP = Bed net polyester fabric. The curves represent the proportion of mosquitoes remaining in the treated chamber (retention) over time, with escape treated as the event. Statistical significance was assessed using the log-rank test.

**Fig 4:**
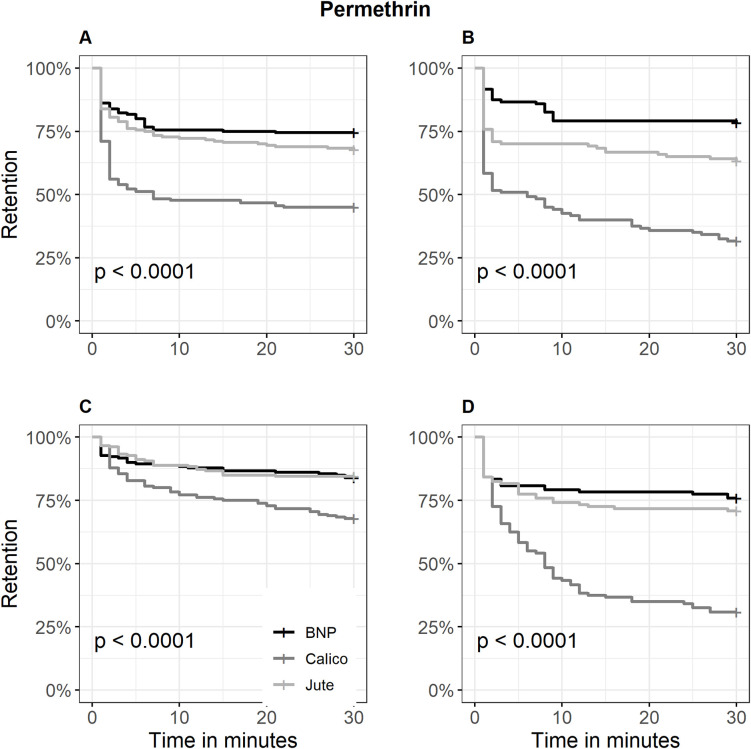
Kaplan–Meier curves for the retention probability of *Aedes aegypti* across three permethrin-treated fabrics. A = Contact retention probability at low dose; B = Contact retention probability at high dose; C = Non-contact retention probability at low dose; D = Non-contact retention probability at high dose; BNP = Bed net polyester fabric. The curves represent the proportion of mosquitoes remaining in the treated chamber (retention) over time, with escape treated as the event. Statistical significance was assessed using the log-rank test.

Kaplan-Meier curves supported these findings (Figs 2–4). Metofluthrin produced early plateauing curves in contact trials at both low and high dose levels (retention: > 85% and 100%, respectively), indicating limited irritancy. In non-contact trials at low dose, retention probability declined gradually, with Calico showing the steepest initial drop reaching 42.2% (57.8% escaped) (Fig 2). With transfluthrin, fabrics showed no significant differences in mosquito retention in contact trials (P = 0.16), with overlapping escape probability curves across Jute and Calico. In contrast, non-contact trials at the high dose showed peak spatial repellency, with retention dropping to 75% within 5 minutes for both fabrics and further declining to 40% (60% escaped) and 36.1% (63.9% escaped), respectively (Fig 3). Permethrin-treated Calico consistently exhibited strongest irritancy and spatial repellency. In contact trials, retention dropped to 50% within 10 minutes at both dose levels, and further declined to 31.7% at the high dose, indicating the strongest overall irritancy (68.3% escaped). In non-contact trials at the high dose, retention decreased to 30.8%, reflecting the strongest overall spatial repellency (69.2% escaped) (Fig 4).

Overall, Calico demonstrated the strongest spatial repellency and irritancy effects against *Ae. aegypti*, while BNP consistently produced the lowest escape rates.

### Toxicity

GLMM analysis showed significant effects on *Ae. aegypti* knockdown and mortality (Table 2). Calico and Jute exhibited significantly reduced odds of knockdown and mortality, highlighting the higher toxicity associated with the reference BNP fabric. With insecticides, the odds were significantly increased under metofluthrin treatment (OR = 32.27; 95% CI: 22.26–46.79) followed by transfluthrin relative to permethrin. Amongst the GLMM interaction terms, the odds of knockdown were significantly increased with Transfluthrin x Calico and Metofluthrin x Calico but reduced with Jute interactions. Between exposure modes, knockdown odds were significantly increased within contact trials (OR = 49.17; 95% CI: 40.02–60.41). Also, the odds were significantly higher at a high dose (OR = 9.32; 95% CI: 7.81–11.12) than low dose. Mortality followed a similar pattern, with significantly reduced odds for Calico and Jute exposure. However, the odds of mortality were significantly elevated under contact exposure (OR = 58.88; 95% CI: 47.27–73.34), metofluthrin treatment (OR = 5.26; 95% CI: 3.78–7.30), high dose (OR = 13.12; 95% CI: 10.96–15.71), and the interaction of both insecticides with cotton (Table 2).

**Table 2:** GLMM estimates of *Aedes aegypti* knockdown at 1-h and mortality at 24-h post exposure to insecticide-treated fabrics.

		Knockdown			Mortality		
Variable		OR	95% CI	p-value	OR	95% CI	p-value
(Intercept)		1	–	–	1	–	–
Fabric	Calico	0.085	0.060–0.120	***	0.062	0.043–0.090	***
	Jute	0.336	0.243–0.464	***	0.225	0.161–0.314	***
Test type	Contact	49.17	40.02–60.41	***	58.88	47.27–73.34	***
Dose category	High	9.316	7.806–11.12	***	13.12	10.96–15.71	***
Insecticide	Metofluthrin	32.27	22.26–46.79	***	5.255	3.783–7.299	***
	Metofluthrin x Calico	3.221	1.968–5.271	***	3.936	2.455–6.309	***
	Metofluthrin x Jute	0.337	0.210–0.541	***	0.150	0.094–0.239	***
	Transfluthrin	1.105	0.815–1.500	0.520	0.239	0.173–0.328	***
	Transfluthrin x Calico	5.021	3.196–7.890	***	4.932	3.040–8.000	***
	Transfluthrin x Jute	0.750	0.485–1.159	0.194	1.091	0.684-1.739	0.716

OR = odds ratio; CI = confidence interval. Odds ratios are relative to the reference levels; OR > 1 indicates increased odds, OR < 1 indicates reduced odds. Interaction terms (e.g., Permethrin x Calico) reflect conditional effects.

### Chemical retention analysis

**Fig 5:**
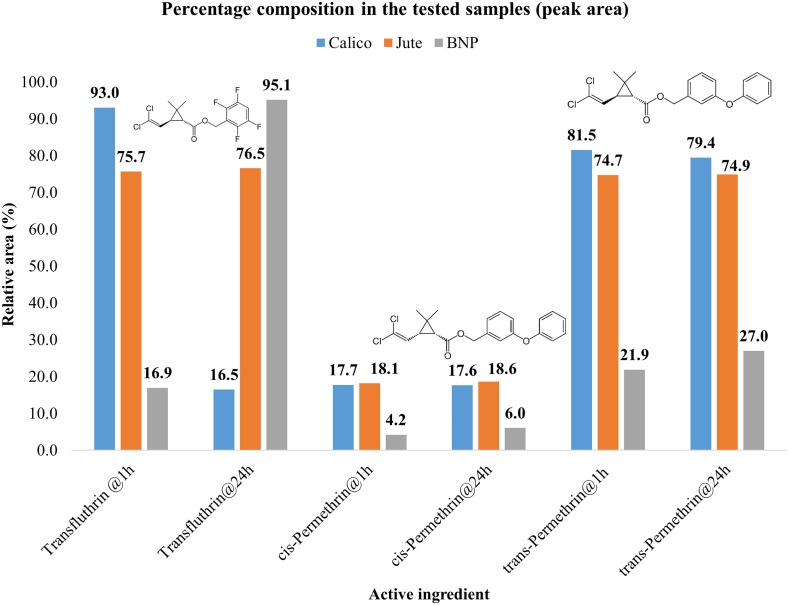
GC–MS retention of transfluthrin and permethrin on fabrics (Calico, Jute, and BNP) at 1h and 24h drying. Retention times: transfluthrin 7.86–7.87 min; cis‑permethrin 22.74 min; trans‑permethrin 23.08–23.09 min.

GC-MS analysis was conducted to assess the retention ability of Jute, Calico and BNP treated with transfluthrin and permethrin after the 1 h and 24 h drying periods. The area composition per sample was calculated for each active ingredient along with the retention times. Transfluthrin concentration was relatively stable in Jute fabric (75.7% at 1 h and 76.53% at 24 h) compared to Calico and BNP which exhibited large differences between the two time periods. The relative area composition of *cis-* and *trans-* permethrin extracted from the three fabric was generally less variable for the two time periods for all fabrics (Fig 5). A slight variation in the retention times of the analyzed compounds was observed between the two drying periods but the same for the tested fabrics.

## Discussion

Insecticide-treated clothing has long served as a frontline defense against arthropod bites, particularly in military and occupational settings [10,20,46]. While permethrin-treated fabrics are well characterized for their dual repellent and insecticidal effects, the role of volatile spatial repellents in treated textiles remains poorly defined. This study used a laboratory-based excito-repellency system to compare the spatial repellency, contact irritancy, and toxicity of three distinct repellent-treated fabrics against *Ae. aegypti*. The results demonstrate that behavioural and toxicological responses varied significantly, highlighting the importance of fabric-insecticide compatibility in determining protective efficacy.

By distinguishing escape responses occuring without contact (spatial repellency) from those occurring after contact (irritancy) [47], this study demonstrated that spatial repellency was generally more pronounced than irritancy (Table 1)—with the notable exception of permethrin-treated fabrics which demonstrated mixed results (Fig 4). This pattern likely reflects the volatile nature of spatial repellents, which activate olfactory-mediated avoidance behaviors prior to contact [5,24]. In field applications, spatial repellency translates into protective zones that reduce human–vector contact [5], especially during daytime or outdoor activities where insecticide-treated nets are impractical. This is critical for *Ae. aegypti*, whose diurnal biting behavior limits the efficacy of traditional bed nets [8]. In contrast, irritancy requires direct tarsal engagement with treated surfaces, a response that may be delayed or suppressed by rapid knockdown [31] due to insecticide toxicity.

Calico consistently produced the highest escape response across trials (Table 1), reinforcing its potential as a high-performance substrate for insecticide-treated clothing. This aligns with findings from a previous study [48], where transfluthrin-treated calico repelled *Ae. aegypti* more effectively than polyester or poplin, achieving up to 70% repellency in high-throughput screening assays. Anuar and Yusof [12] reviewed mosquito-repellent textiles and noted that natural fibres like Calico tend to retain repellent agents more effectively than synthetic blends, such as BNP, due to their hydrophilic nature and fibre porosity. The enhanced efficacy of Calico may be attributed to its physical and chemical properties. As a plain-woven, unbleached cotton fabric, Calico offers a porous, hydrophilic surface that facilitates deeper absorption and slower release of active ingredients especially for compounds like transfluthrin and permethrin [49]. Jute demonstrated comparable spatial repellency to Calico when treated with transfluthrin at a high dose (Fig 3), suggesting that its coarse, fibrous structure may facilitate effective volatilization of highly active compounds. Unlike Calico, which maintained repellency across insecticides and doses, Jute’s performance was inconsistent—highlighting the importance of fabric–insecticide compatibility. Previous studies have demonstrated that transfluthrin-treated Jute strips reduced mosquito human landing in semi-field trials, but emphasized the need for high-dose formulations to achieve consistent protection [14,50]. These findings suggest that while Jute may serve as a viable alternative to cotton in spatial repellent applications, its efficacy is contingent on the volatility and concentration of the active ingredient. Operationally, Jute could be prioritized for high-dose transfluthrin formulations in outdoor or semi-enclosed settings [18], whereas Calico offers broader versatility across insecticide classes and exposure modes.

Toxicity estimates from the present study revealed that contact exposure increased knockdown and mortality by 49.2 and 58.9 times, respectively, compared to non-contact trials. Notably, metofluthrin-treated fabrics produced 32.27 times higher knockdown and 5.26 times greater mortality than permethrin-treated baseline (Table 2). These findings are consistent with Ritchie and Devine [51], who reported 80–90% mortality in rooms treated with polyethylene emanators impregnated with 5–10% metofluthrin. Similarly, Kim et al [52] observed high knockdown and mortality using metofluthrin-treated filter papers against *Ae. aegypti*, reinforcing its potent toxicological profile. Excito-repellency assays by Sukkanon et al [31] and Yan et al [16] further support these observations, showing that transfluthrin-treated papers induced high knockdown and reduced escape rates in both *Ae. aegypti* and *Anopheles minimus*. These results suggest that toxicity can mask irritancy where rapid incapacitation limits the opportunity for escape. In our study, spatial repellency was also affected by toxicity at high doses, indicating a complex interplay between dose, volatility, and fabric-mediated release. The exception with permethrin-treated fabrics likely reflects its low volatility and strong contact irritancy, which relies on tactile stimulation rather than airborne dispersion [10]. This “hot foot” effect is well-documented in contact bioassays [53], but its spatial efficacy remains limited unless co-formulated with volatile compounds [54]. Among the fabrics, reduced odds of both knockdown and mortality for Jute and Calico compared to BNP are not explained in the present study. However, this reflects the hydrophobic and low-porosity surface that retains pyrethroids at the fiber–air interface, enhancing both contact dose and near-surface availability, whereas cotton and jute sequester insecticide within the fiber matrix, reducing immediate bioavailability [55]. These findings underscore the importance of differentiating repellent modes of action and optimizing formulations based on intended use—whether for spatial protection, contact deterrence, or toxicity-driven control.

The present study findings were reinforced by the GC-MS analysis results (Fig 5), which revealed fabric-specific retention dynamics for transfluthrin and permethrin. Volatile repellent-treated fabrics have been observed to lose efficacy as time passes [56,57], a factor we hypothesised could have led to the variations in our results after air-drying the fabrics for 24 h before each bioassay. Permethrin concentration was relatively the same across the 24 h period for all the three fabrics. Sullivan et al [57] reported a similar result where they observed unchanged absorption of permethrin on calico (65%)/polyester (35%) fabrics for one week and one month post-treatment. The stable retention in Calico likely contributed to its superior performance in both irritancy and spatial repellency trials. In contrast, transfluthrin—a highly volatile pyrethroid—exhibited fabric-dependent retention dynamics. Its concentration remained stable on Jute but varied significantly on Calico and BNP between the two drying periods. These fluctuations were inconsistent and did not follow a predictable decay pattern, suggesting that fabric structure and chemical affinity may influence volatilization and bioavailability [13]. Comparable observations were reported by Ogoma et al [56], who found undetectable airborne transfluthrin at 1 h but variable concentrations at 24 h in rooms treated with jute strips, despite identical treatment protocols.

This study has several limitations that should be acknowledged. First, metofluthrin was excluded from the GC–MS analysis due to logistical constraints, which limited direct comparison across all tested insecticides. Second, knocked‑down mosquitoes were included in the escape analysis, and because escape probability was calculated relative to the total number at the end of the bioassay, this may have underestimated behavioral responses. Finally, bulk permethrin was used in excito-repellency assays rather than separating cis‑ and trans‑isomers, which may have masked potential differences in isomer‑specific efficacy. These limitations highlight the need for future studies incorporating extended sampling intervals, isomer‑specific analyses, and refined behavioral endpoints to strengthen the interpretation and application of insecticide‑treated fabrics in mosquito control.

## Conclusion

This study demonstrates that volatile pyrethroid-treated fabrics particulary cotton substrates treated with transfluthrin, can provide strong spatial repellency against *Ae. aegypti,*while polyester substrates produce greater toxic effects. Repellent-treated fabrics offer a promising complement to *Ae. aegypti* control strategies due to their affordability and ease of deployment. Our findings demonstrate that insecticide-treated fabrics can serve as effective protective barriers—whether as clothing, house screens, bed nets, or curtains—to reduce human–vector contact. This can help in interrupting mosquito-borne disease transmission. Such applications align with WHO and EPA recommendations on insecticide-treated nets and treated materials as core vector control interventions [9,58]. However, operational use requires careful consideration of safety and sustainability: insecticide exposure on skin needs careful monitoring, efficacy declines with repeated washing, warranting periodic re-evaluation, resistance management strategies should be implemented, and disposal practices must avoid environmental contamination [58].
